# Tropical surface temperature response to vegetation cover changes and the role of drylands

**DOI:** 10.1111/gcb.16455

**Published:** 2022-10-11

**Authors:** Andrew F. Feldman, Daniel J. Short Gianotti, Jianzhi Dong, Isabel F. Trigo, Guido D. Salvucci, Dara Entekhabi

**Affiliations:** ^1^ Biospheric Sciences Laboratory NASA Goddard Space Flight Center Greenbelt Maryland USA; ^2^ NASA Postdoctoral Program NASA Goddard Space Flight Center Greenbelt Maryland USA; ^3^ Department of Civil and Environmental Engineering Massachusetts Institute of Technology Cambridge Massachusetts USA; ^4^ Instituto Português do Mar e da Atmosfera I.P. (IPMA) Lisbon Portugal; ^5^ Instituto Dom Luiz (IDL) Lisbon Portugal; ^6^ Department of Earth and Environment Boston University Boston Massachusetts USA

**Keywords:** biophysical feedbacks, drylands, land surface temperature, satellite remote sensing, surface albedo, tropical vegetation

## Abstract

Vegetation cover creates competing effects on land surface temperature: it typically cools through enhancing energy dissipation and warms via decreasing surface albedo. Global vegetation has been previously found to overall net cool land surfaces with cooling contributions from temperate and tropical vegetation and warming contributions from boreal vegetation. Recent studies suggest that dryland vegetation across the tropics strongly contributes to this global net cooling feedback. However, observation‐based vegetation‐temperature interaction studies have been limited in the tropics, especially in their widespread drylands. Theoretical considerations also call into question the ability of dryland vegetation to strongly cool the surface under low water availability. Here, we use satellite observations to investigate how tropical vegetation cover influences the surface energy balance. We find that while increased vegetation cover would impart net cooling feedbacks across the tropics, net vegetal cooling effects are subdued in drylands. Using observations, we determine that dryland plants have less ability to cool the surface due to their cooling pathways being reduced by aridity, overall less efficient dissipation of turbulent energy, and their tendency to strongly increase solar radiation absorption. As a result, while proportional greening across the tropics would create an overall biophysical cooling feedback, dryland tropical vegetation reduces the overall tropical surface cooling magnitude by at least 14%, instead of enhancing cooling as suggested by previous global studies.

## Graphical abstract

With limited study of biophysical feedbacks on temperature in the tropics, especially in drylands, we use multiple satellite platforms to determine the influence of tropical dryland plants on surface temperature. We find that while vegetation across the tropics tends to net cool the surface, dryland plants reduce this tropical cooling by at least 14%, contrary to enhanced cooling found in some previous studies. The dryland cooling reduction is attributed to their plants' reduced evaporation due to aridity and proportionally greater vegetal absorption of solar radiation.

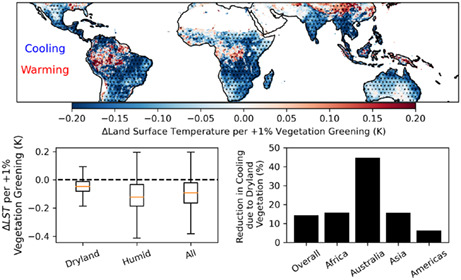

## 1 INTRODUCTION

Vegetation's effect on the surface energy balance remains an open question due to its competing influences on land surface temperature (Shen et al., 2015). It is essential to determine vegetation's net effect on the energy balance given ongoing global land cover changes, including deforestation, agricultural expansion, climate change‐induced greening, and replanting efforts (Alkama et al., 2022; Bonan, 2008; Bright et al., 2017; Jackson et al., 2008; Rigden & Li, 2017). Plants have a strong influence on the energy balance and evapotranspiration estimation, as shown at interannual timescales (Li et al., 2013). Climate model projections are also highly sensitive to vegetation biophysical parameterization (Devaraju et al., 2015; Feddema et al., 2005). Without accurate characterization of these biophysical processes, climate model prediction uncertainty of the land surface state increases, which could cause targeted land cover mitigation efforts like reforestation to have unintended consequences of enhancing global warming (Arora & Montenegro, 2011; Bala et al., 2007; Betts, 2000).

Net vegetation effects on the surface energy balance differ across the globe (Alkama et al., 2022; Duveiller et al., 2018). The current consensus is that vegetation globally creates net cooling feedbacks (Zeng et al., 2017). This cooling signal is dominated by a greater fraction of vegetation cover (FVC) in temperate and tropical environments net cooling through enhanced turbulent energy dissipation from greater surface roughness and transpiration (Bounoua et al., 2000; Juang et al., 2007; Luyssaert et al., 2014; Tang et al., 2018). This mid‐to‐lower‐latitude vegetation net cooling feedback is exemplified by findings of tropical deforestation enhancing surface warming (Alkama & Cescatti, 2016; Mahmood et al., 2014; Silvério et al., 2015; Vargas Zeppetello et al., 2020). These biophysical cooling effects are partially negated by net warming effects of boreal vegetation due to a dominant surface albedo effect of proportionally greater vegetal absorption of solar radiation (Lee et al., 2011; Li et al., 2015; Peng et al., 2014).

Conceptual understanding of vegetation's influence on the energy balance can be developed, for example, using the force restore model where the modeled rate of land surface temperature (LST) change, or *d*(LST)/*dt*, increases with higher net radiation and decreases with turbulent fluxes (Deardorff, 1978). From this model, several pathways of vegetation influence on *d*(LST)/*dt* are apparent. (1) A warming effect exists where more vegetation cover decreases surface albedo and increases net surface radiation. (2) A cooling effect exists where more vegetation cover increases surface roughness and conductance, which enhances turbulent energy flux dissipation (Chen et al., 2020; Margulis, 2017). (3) A cooling effect exists where more vegetation cover enhances transpiration of water from deeper soil layers than accessible from bare soil evaporation (Katul et al., 2012). Rather than present an exhaustive list of mechanisms, we emphasize that these mechanisms compete and are sensitive to model parameterization choices.

A model‐based study necessarily leads to uncertainty because of the need for model parameterizations linking vegetal effects to surface temperature. Specific parameters must be estimated to represent, for example, stomatal regulation and transpiration response to soil and atmosphere conditions, surface albedo sensitivity to vegetation characteristics, and vegetation interactions with the boundary layer (e.g., Rotenberg & Yakir, 2010). Both parameter uncertainty and model structural uncertainty will consequently create uncertain and inconsistent biophysical parameterizations between models (Pitman et al., 2009). Observational studies are thus needed to constrain and benchmark the results of these complex representations of vegetal effects on surface temperature.

Recent global studies suggest that tropical drylands enhance net cooling feedbacks under global greening (Alkama et al., 2022; Forzieri et al., 2018). However, theoretical considerations and sparse field studies call into question findings of strong vegetal surface cooling in semi‐arid locations: limited water‐availability will reduce evaporation, the surface's main mechanism of removing energy in these warm, dry environments (Javadian et al., 2022; Li et al., 2014; Rotenberg & Yakir, 2010). Specifically, given that latent heat flux becomes an important control on the surface energy balance in warmer conditions (Bateni & Entekhabi, 2012), soil moisture losses will likely subdue vegetal cooling via reduced evaporation in tropical water‐limited environments (Seneviratne et al., 2010). Model‐free, observation‐based studies mainly investigate these processes in the boreal and mid‐latitudes, but are limited in the tropics. There are also limited efforts to identify mechanistic drivers that would explain spatial patterns of biophysical feedbacks.

In this study, we use satellite observations to isolate and attribute the effect of vegetation cover on LST, via the diurnal rate of land surface warming. We ask: (a) does more vegetation cover net cool the surface across the tropics, especially in drylands? (b) Which surface energy balance mechanisms are responsible for the observed spatial pattern of vegetal effects on surface temperature in the tropics? (c) To what degree does tropical dryland vegetation net warm or cool the land surface compared to the remainder of the vegetated tropics?

Previous studies address components of our research questions with LST (Chen et al., 2020; Forzieri et al., 2018). A novel feature of our analysis is that we first establish connections between the diurnal rate of temperature change (*d*(LST)/*dt*) and FVC using geostationary satellite observations in Africa, rather than temperature magnitude itself. This is because LST is a state variable and thus depends on and is confounded by coupling with other land surface states. The LST time derivative largely removes state dependencies. *d*(LST)/*dt* also more closely relates to surface energy flux components, where increased *d*(LST)/*dt* indicates reduced latent heat flux (Bateni & Entekhabi, 2012; Deardorff, 1978). As such, detecting a variable's impact (i.e., vegetation cover) on *d*(LST)/*dt* more confidently establishes its direct influence on land surface temperature than does the same variable's statistical connection to LST. Using independently observed variables also should, in principle, provide a better estimate of the true linkage between the variables because they do not contain confounding imposed parameterizations as found in models. For example, recent work found biases in model reanalysis diurnal temperature behavior and its interactions with vegetation compared to in‐situ measurements (Panwar & Kleidon, 2022). For these reasons, diurnal temperature observations have been previously used to establish surface energy balance interactions with vegetation and the atmosphere, though for different research questions (Dai et al., 1999; Feldman et al., 2022, 2019; Panwar et al., 2020; Panwar & Kleidon, 2022).

## 2 MATERIALS AND METHODS

### 2.1 Workflow summary

We addressed our research questions with three main assessments. Assessment I determined the observed African vegetation‐temperature relationships. Specifically, a geostationary satellite located over Africa provides sub‐hourly sampling of various land surface variables, which was used to holistically investigate linkages between vegetation and temperature. Assessment II uses these same datasets to determine mechanistic drivers that describe spatial patterns of results in Assessment I. Assessment III uses global satellite retrievals to evaluate whether the patterns of LST response to vegetation variability found in the Africa‐only analysis hold across the tropics.

Ultimately, our approach presents confidence in patterns of vegetation influence on the surface energy balance for several reasons. (a) The use of observations here is an advantage given difficulty with modeling competing effects of vegetation cover on the surface energy balance and allows independent assessment of global model outputs. (b) Assessment I includes several regressions to gain confidence in patterns of results. (c) Assessment II explains the patterns with identification of observed drivers. (d) Assessment III uses satellite observations independent from the other assessments, providing confidence in findings described in Assessments I and II.

**FIGURE 1:**
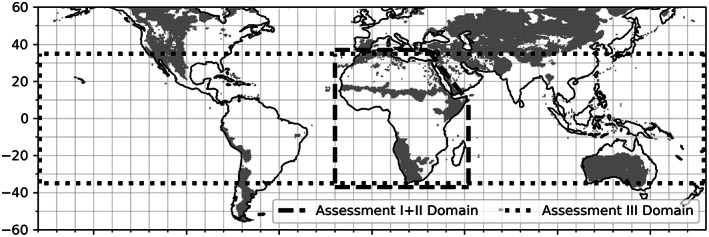
Map of the study domain. Assessments I and II occur in Africa. Assessment III occurs on vegetated landscapes between 35°S and 35°N. all vegetated land surfaces are evaluated within the domains. For reference, gray shading denotes vegetated drylands based on CPC total annual rainfall of less than 500 mm. Map lines delineate study areas and do not necessarily depict accepted national boundaries.

The datasets used in both analyses are shown in Table 1 with details provided in Section 2.2. The study domain is shown in Figure 1. To evaluate our research questions, we contrasted drylands against more humid regions using a standard definition of land surfaces receiving less than 500 mm of rainfall (Noy‐Meir, 1973). Africa was chosen for Assessment I not only because of extensive sub‐hourly satellite sampling of land surface conditions but also for its large area of vegetated drylands.

**TABLE 1:** Datasets and their use within each assessment

	Variable	Instrument/dataset	Reference	Dataset use notes
Assessment I: African Vegetation‐Temperature Interactions	Fraction of Vegetation Cover (FVC)	SEVIRI	Trigo et al. (2011)	Assess annual mean vegetation cover response to surface temperature
	Land Surface Temperature (LST)	SEVIRI	Trigo et al. (2011)	
	Downward Surface Solar Radiation (R_S_)	SEVIRI	Trigo et al. (2011)	Control for light availability
	Soil Moisture (θ)	SMAP	Feldman et al. (2021)	Control for soil water availability
	Precipitation (P)	CHIRPS	Funk et al. (2015)	Rainfall gradient analysis; robustness test
	Land Cover Classification	IGBP	Kim et al. (2013)	Vegetation type analysis
Assessment II: Africa Mechanism Identification	Fraction of Vegetation Cover (FVC)	SEVIRI	Trigo et al. (2011)	Seasonal analysis; Surface albedo response
	Land Surface Temperature (LST)	SEVIRI	Trigo et al. (2011)	Seasonal analysis, Energy dissipation efficiency
	Downward Surface Solar Radiation (R_S_)	SEVIRI	Trigo et al. (2011)	Seasonal analysis, Control for light availability
	Soil Moisture (θ)	SMAP	Feldman et al. (2021)	Seasonal analysis, Control for water availability
	Vapor Pressure Deficit (VPD)	AIRS	Teixeira (2013)	Seasonal analysis
	Surface Albedo (α)	SEVIRI	Trigo et al. (2011)	Surface albedo response
Assessment III: Tropical Vegetation‐Temperature Interactions	Land Surface Temperature (LST)	MODIS	Wan et al. (2015)	Assess interannual vegetation cover response to surface temperature
	Normalized Difference Vegetation Index (NDVI)	MODIS	Didan et al. (2021)	
	Downward Surface Solar Radiation (R_S_)	MERRA2	Gelaro et al. (2017)	Control for light availability
	Precipitation (P)	CPC	Chen et al. (2008)	Control for water availability
	Land Cover Classification	IGBP	Kim et al. (2013)	Remove bare soil regions
	Leaf Area Index (LAI)	MODIS	Myneni et al. (2021)	Alternative test

*Note*: Assessments I and II were carried out over Africa at a 9 km grid scale. The temporal resolution varies per analysis (see Sections 2.3 and 2.4). Assessment III was carried out across the tropics (between 35°S and 35°N), at a half degree grid scale, and at an interannual timescale.

### 2.2 Datasets

For Assessment I (the Africa‐only analysis), we used several retrievals from the Spinning Enhanced Visible and Infrared Imager (SEVIRI), which is on‐board the European and EUMETSAT space agency's Meteosat Second Generation geostationary satellite series and is at a 3 km resolution (Trigo et al., 2011). SEVIRI is geostationary with its highest quality measurements in Africa. Its 15‐min temporal resolution allows more frequent samples of land surface conditions under cloud cover than global, low‐Earth orbit satellites. LST is sampled at 15‐min time steps and is sensitive to soil‐vegetation temperature, which describes the surface energy balance more than air temperature (Panwar et al., 2019). We also used SEVIRI‐retrieved FVC, and downwelling surface shortwave radiation (R_S_) to partition the effects of vegetation on the *d*(LST)/*dt* signal (Carrer et al., 2019). Soil moisture (θ) from NASA's Soil Moisture Active Passive (SMAP) satellite was used at a 9 km grid with 1–3 day sampling using a retrieval algorithm that is independent from vegetation cover (Feldman et al., 2021). These datasets were all regridded to a 9 km Equal Area Scalable Earth‐2 (EASE2) grid. Only data from 2018 were used in the main assessment of relationships, though SEVIRI data from 2004 to 2019 were used in a robustness test (see SI). For this auxiliary robustness test, Climate Hazards Infrared Precipitation with Stations (CHIRPS) precipitation was used in place of soil moisture to control for annual moisture availability changes over 2004 to 2019 (Funk et al., 2015), given that SMAP is only available after 2015. International Geosphere‐Biosphere Programme (IGBP) classifications are used to evaluate differences in behavior with vegetation type (Kim, 2013).

To identify mechanistic drivers of spatial patterns in Assessment I, Assessment II used all the same variables as Assessment I (Table 1). It additionally included daily SEVIRI retrievals of surface albedo (α) and NASA's Atmospheric Infrared Sounder (AIRS) retrievals of vapor pressure deficit (VPD) in the boundary layer (at 850mb) (Teixeira, 2013). Analysis of the effects of seasonal changes in aridity used SEVIRI LST, FVC, and R_S_ in addition to SMAP θ and AIRS VPD. Determination of energy dissipation efficiency uses 15‐min increments of SEVIRI LST. Finally, the surface albedo‐FVC interaction analysis used SEVIRI FVC and α while using SMAP θ and SEVIRI R_S_ to control for water and light availability.

To evaluate whether the effects of vegetation cover on the surface energy balance determined in Africa occur across all tropical regions (Assessment III), we used annual mean NDVI (MOD13C1) and LST (MYD11C2) from MODIS Terra and Aqua instruments respectively (Didan, 2021; Wan et al., 2015). NOAA Climate Prediction Center (CPC) total annual precipitation and MERRA2 surface incoming shortwave flux were used to control for annual mean energy and water availability, respectively (Chen et al., 2008; Gelaro et al., 2017). CPC was used here instead of CHIRPS to maintain independence from Assessment I and II. MODIS Aqua LST retrievals are at approximately 1:30 pm local time. Additional analyses were performed using leaf area index (LAI) from MODIS to assess how their use may alter results (Myneni et al., 2021). All datasets were acquired over their co‐occurring span of 19 years between 2003 to 2021 and were regridded to 0.5 degrees.

### 2.3 Assessment I: African vegetation‐temperature interactions

#### 2.3.1 Assessment I statistical analysis

SEVIRI 15‐min LST from 7 to 11 am local solar time were used to calculate the median daily *d*(LST)/*dt*, which is directly related to the diurnal temperature range. Sub‐daily *d*(LST)/*dt* was computed by differencing each time adjacent LST value between 7 am and 11 am and dividing by their time between increments. Two time adjacent LST increments are typically differenced and divided by 15 min, but sometimes over longer time spans given quality flags (i.e., cloud cover). For each day, the median was taken of all available sub‐daily increments and units converted to K/h to obtain a daily time series of *d*(LST)/*dt*. The median 7 to 11 am *d*(LST)/*dt* integrates the diurnal LST cycle while being less sensitive to peak LST timing and to cloud coverage than more common methods that subtract morning and afternoon temperature snapshots (Holmes et al., 2015). Namely, it considers the time range when LST is consistently rising nearly linearly before slowing its rise near the daily LST peak (Figure S1). Nevertheless, the *d*(LST)/*dt* median is consistently related to the diurnal temperature range where spatial correlations across Africa between median *d*(LST)/*dt* and the 1:30 pm and 6:00 am LST difference tend to be above 0.8 on a given day. Use of diurnal temperature range for such an application is likely still acceptable in the absence 15‐min data.

For our primary approach to partition FVC's effect on *d*(LST)/*dt*, we used spatial conditioning to bin pixels of nearly identical long term mean soil moisture (±0.0025 m^3^m^−3^) and mean incoming solar radiation (±1.25 Wm^−2^). For the pixels within each bin, we applied

(1) $$E\left[\frac{d\left(\mathrm{LST}\right)}{\mathrm{dt}}\right]=\beta_{0}+\beta_{\mathrm{FVC}}E\left[\mathrm{FVC}\right]+\varepsilon ,$$

*d*(LST)/*dt* and FVC annual means in 2018 were used in Equation 1. β_0_ and ε are the y‐intercept and residual, respectively. β_FVC_ represents the linear effect of FVC on *d*(LST)/*dt*. Statistically significant (*p* < .05) positive and negative values of β_FVC_ indicate a warming and cooling effect of vegetation, respectively. Statistically insignificant values of β_FVC_ are considered neutral effects here. This approach assumes a space–time equivalence where changes in annual means of FVC and *d*(LST)/*dt* between pixels with similar water and energy availability are assumed to also occur in time at a given location. Pixels with mean SEVIRI FVC of zero were removed from the analysis.

We focus on this approach for several reasons. (a) Use of median *d*(LST)/*dt* can reveal more causal relationships between vegetation cover and LST than can direct statistical relationships between FVC and LST. (b) It uses 15‐min LST observations from the SEVIRI geostationary satellite, which provide greater temporal coverage under cloud cover conditions given their more frequent sub‐daily sampling than satellites with global coverage. (c) The approach attempts to determine long‐term, climatic relations between variables that are indicative of how they would co‐vary at annual and decadal timescales relevant to land cover changes. Indeed, differences in climatic, edaphic, and topographic conditions across space may confound $\beta_{\mathrm{FVC}}$ interpretations from Equation 1 of how annual *d*(LST)/*dt* and FVC vary in time at a given location. However, we expect that this spatial approach will consider effects of beyond‐decadal scale feedbacks and ecosystem equilibrium states (Eagleson & Segarra, 1985) that may not appear in sub‐annual and interannual timescale regression analyses. Additionally, the use of water and light availability to bin pixels is expected to provide similarity between ecosystems such that their spatial vegetation‐temperature covariations are more likely to reflect those in time at a location than differences in climate.

#### 2.3.2 Assessment I robustness tests

We conducted several tests to evaluate the robustness of the Equation 1 approach as well as to interpret its results. These tests were also meant to motivate the analysis of vegetation‐temperature interactions across the tropics in Assessment III using interannual variability of global satellite datasets. More details about the tests can be found in the SI.

Tests were performed to determine whether the spatial relationships resulting from Equation 1 also occur in time. We focused our tests on longer timescale, interannual variations because of our interest in biophysical feedbacks under climate change. 16 years of SEVIRI diurnal temperature range observations (2004–2019) in each pixel were used to determine whether the spatial relationships from Equation 1 also hold in time at interannual timescales (see SI). Additionally, four years (2015–2019) were used for four randomly selected pixels in space within each bin to determine the combined effect of interannual and spatial variability (see SI). Rather than the full diurnal temperature cycle, this analysis used the difference in 1:30 pm and 6:00 am LST, assumed to be the maximum and minimum daily temperature (Feldman et al., 2019). The interannual timescale analysis ultimately has uncertainties due to small sample size of 16 data points per pixel as well assumptions of timing of maximum and minimum LST. Nevertheless, these tests were carried out for qualitative comparison with those from Equation 1 in Section 2.3.1.

It is also expected that there will be differences in determining temperature‐vegetation relationships from daily variations, given strong confounding effects between land surface variables at seasonal timescales. We performed a panel regression that partitions the sub‐annual and interannual scale interactions between FVC and *d*(LST)/*dt* (see SI). Seasonal and intra‐seasonal regressions are performed for comparison (see SI).

The spatial analysis in Equation 1 was repeated using the mean annual afternoon temperature in place of mean annual *d*(LST)/*dt* to evaluate the connection of the *d*(LST)/*dt* results to LST itself. The mean daily peak temperature is approximated as the mean temperature between 12:30 pm and 2:30 pm (Figure S1).

### 2.4 Assessment II: Mechanisms driving African vegetation‐temperature interactions

Several analyses were conducted here to attribute spatial variations in $\beta_{\mathrm{FVC}}$ to mechanistic drivers. Namely, we tested whether aridity, energy dissipation efficiency, and surface radiation absorption describe why vegetation shifts its control on surface temperature along climatic gradients, especially in drylands compared to more humid environments.

We first evaluated the interaction of FVC and *d*(LST)/*dt* during different seasons to infer the interactive effects of radiation and moisture availability. Specifically, the spatial timescale analysis in Equation 1 is repeated in the halves of the year with low and high vegetation cover, defined as times of year below and above the FVC median, respectively,

(2) $$E\left[d\left(\mathrm{LST}\right)/\mathrm{dt}\right]=\beta_{0}+\beta_{\mathrm{FVC}}E\left[\mathrm{FVC}\right]+\beta_{\theta }E\left[\theta \right]+\beta_{R_{S}}E\left[R_{S}\right]+\varepsilon .$$

Equation 2 was performed as in Equation 1 with binning based on water and light availability, but the seasonal means of R_S_ and θ under seasons of high and low vegetation coverage are included as regressors in Equation 1 to control for the direct effects of the environmental factors on LST that do not necessarily involve the influence of vegetation. As such, we expect that differences in *β*_FVC_ between seasons of low and high vegetation cover can be interpreted as effects of climatic factors (R_S_, θ, and VPD) on FVC‐*d*(LST)/*dt* interactions. Comparability of *β*_FVC_ between seasons is possible because *β*_FVC_ is based on unit changes in FVC, not its overall magnitude. We do not aim to determine effects within specific seasons, but rather test whether FVC‐*d*(LST)/*dt* interactions change with seasons and identify environmental factors that may be driving these changes. Seasons of high and low vegetation coverage are naively chosen to test how different environmental conditions seasonally limit photosynthesis rather than imposing only water‐limitation, for example, from wet and dry season definitions. We computed the seasonal differences in β_FVC_ and related them to the seasonal mean changes in soil moisture, solar radiation, and vapor pressures deficit.

To evaluate strength of energy dissipation through latent and sensible heat and radiation fluxes for different locations, Bateni and Entekhabi (2012) show analytically that *d*(LST)/*dt* can be predicted by LST dependent terms that are all dissipative through surface energy fluxes. As such, the *d*(LST)/*dt* versus LST at hourly timescales is an intrinsic landscape property:

(3) $$\frac{d\mathrm{LST}}{\mathrm{dt}}=\beta_{0}+-\beta_{\mathrm{Eff}}\mathrm{LST}+\varepsilon$$

where *β*_Eff_ describes the efficiency of total land surface energy dissipation. Equation 3 was computed per pixel using available 15‐minute *d*(LST)/*dt* increments and concurrent LST magnitude. *β*_Eff_ is normalized to be unitless by a multiplication factor of 32.5 used in Bateni and Entekhabi (2012) (see their Equation 12).

To quantify whether vegetation fraction has differential effects on surface albedo across Africa, we computed

(4) $$E\left[\alpha \right]=\beta_{0}+\beta_{\alpha ,\mathrm{FVC}}E\left[\mathrm{FVC}\right]+\varepsilon .$$

Equation 4 was repeated identically to Equation 1 in each bin of mean moisture and solar radiation. A higher *β*_α,FVC_ magnitude suggests that changes in vegetation cover have a stronger influence on the vegetated surface's ability to absorb incoming radiation. Note that SEVIRI FVC and surface albedo retrieval processes are independent and use different electromagnetics models (García‐Haro & Camacho, 2014).

To isolate the effects of tropical drylands across these mechanistic analyses, we binned regions with less than and greater than 500 mm of annual total CHIRPS precipitation. We found overall results did not change in varying this dryland threshold by ±200 mm/year.

### 2.5 Assessment III: Tropical vegetation‐temperature interactions

We repeated the analysis using MODIS NDVI and LST observations, but across the vegetated tropics. We only assess the tropics and subtropics (vegetated land surfaces within 35°S to 35°N degrees latitude) given the strong control of water and energy availability in the tropics relevant to the mechanisms discussed in Africa and due to different mechanistic processes (i.e., snow cover) that occur in the mid‐ and high latitudes.

A per‐pixel regression was performed on annual mean values to determine the partial control of NDVI on LST at interannual timescales:

(5) $$E\left[\mathrm{LST}\right]=\beta_{0}+\beta_{\text{NDVI}}E\left[\text{NDVI}\right]+\beta_{P}E\left[P\right]+\beta_{R_{S}}E\left[R_{S}\right]+\varepsilon$$

Variables used in Equation 5 are shown in Table 1. We evaluated the direct statistical connection of vegetation cover to LST here, instead of *d*(LST)/*dt*, where we later argue that there is a causal interpretation of these relationships. NDVI is approximately linearly related to FVC especially in non‐forested regions, which cover most of the tropics by area (Carlson & Ripley, 1997; Fan et al., 2009). As such, we expect some transferability between SEVIRI FVC‐based and MODIS NDVI‐based results. Statistically significant linear trends in annual means were removed from LST and NDVI to remove confounding effects of LST on NDVI, which mainly influenced tropical forest pixels. We multiplied *β*_NDVI_ by 1% of mean annual NDVI between 2003 to 2021 to determine by how much LST would change if mean NDVI were increased by 1% everywhere (denoted as ΔLST). To assess the role of drylands on the LST changes due to biophysical feedbacks of vegetation cover increases, spatial distributions of this ΔLST with and without drylands included were compared using *t*‐tests.

## 3 RESULTS

### 3.1 Assessment I: Relationship between FVC and *d*(LST)/*dt*

**FIGURE 2:**
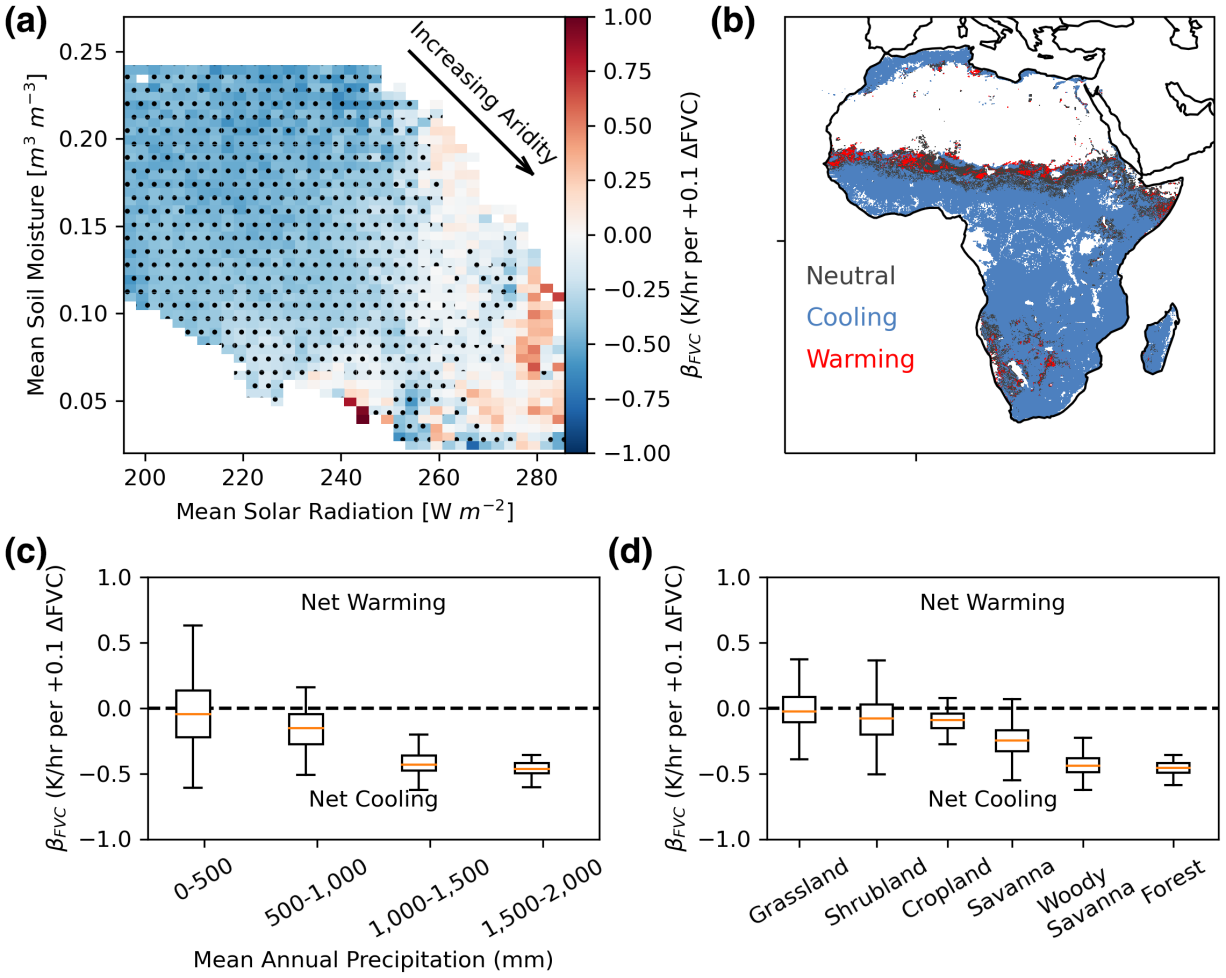
While fraction of vegetation cover (FVC) and rates of diurnal temperature change (*d*(LST)/*dt*) are negatively related (vegetal cooling effects) under most conditions, this relationship becomes subdued or positive (vegetal warming effects) in drier locations. (a) *β*_FVC_ estimated using Equation 1 with a spatial conditioning approach, evaluating interannual and longer timescales while controlling for moisture and energy availability. Values are normalized considering a 0.1 absolute increase in FVC in all locations. Negative (blue) values indicate that FVC reduces *d*(LST)/*dt* (cooling effect). Stippling indicates a statistically significant (*p* < .05) negative *β*_FVC_. Statistically significant positive *β*_FVC_ are not stippled. (b) Locations where significant net warming, neutral, and cooling effects of vegetation are found. (c) Values in (a) binned based on total annual rainfall. (d) Values in (a) binned based on IGBP land cover classifications.

FVC tends to reduce the land surface warming rate (*d*(LST)/*dt*), or net cool, across most of Africa's vegetated biomes (Figure 2). Net cooling effects are detected in 81% of Africa's vegetated surfaces. Over an 8‐h diurnal warming cycle in locations where these net cooling effects are detected, a 10% increase in vegetation cover would suppress the LST diurnal range by about 4 K.

Vegetation cools the surface less when water is limiting (Figure 2). Vegetation cover's influence on *d*(LST)/*dt* gradually transitions from net cooling to net neutral and even net warming from energy‐limited to water‐limited locations. This gradient of reduced net cooling effects can be seen with decreasing annual total rainfall and shorter, less woody vegetation (Figure 2c,d). Locations where neutral or warming effects occur at beyond‐annual timescales are typically water‐limited, characterized by low total rainfall (average of 400 mm/year) and dominant grass and shrubland land cover (Figure S2). Overall, 16% of the vegetated African land surface has no significant (neutral) net effect and 3% has a significant net warming effect (*p* < .05). Similar results are obtained when repeating the analysis on the afternoon 13:30 local temperatures only and less so on morning 6:00 temperatures, suggesting that these vegetal effects impact the diurnal temperature range mainly through daily afternoon LST (Figure S3).

The interannual variability analyses give evidence that the spatial relationships in Figure 1 do occur in time, at least at interannual timescales (Figure S4). Though less certain given low sample size of 16 data points per pixel, the interannual relationships qualitatively show a gradient of reduced net cooling effects of FVC on *d*(LST)/*dt* with more water‐limitation, especially from sub‐humid to arid conditions. These interannual relationships give credence to our space‐for‐time assumptions, or the expectation that spatial variations in annual means within the bins translate to changes in annual means in time at a given location. Furthermore, the panel regression tests and regressions at sub‐annual timescales show that stronger cooling effects are found at sub‐annual scales than at longer timescales (Figures S5 and S6). Moreover, our tests using the panel regression approach indicate that the sign of interactions between FVC and the surface energy balance can switch between intra‐annual and beyond annual timescales, especially in drier environments (Figure S5). Nevertheless, the sub‐annual timescale analysis still shows a reduction of net cooling effects in more water‐limited locations (Figure S6).

### 3.2 Assessment II: Drivers of vegetation effects on the surface energy balance

#### 3.2.1 Vegetation interaction with aridity

**FIGURE 3:**
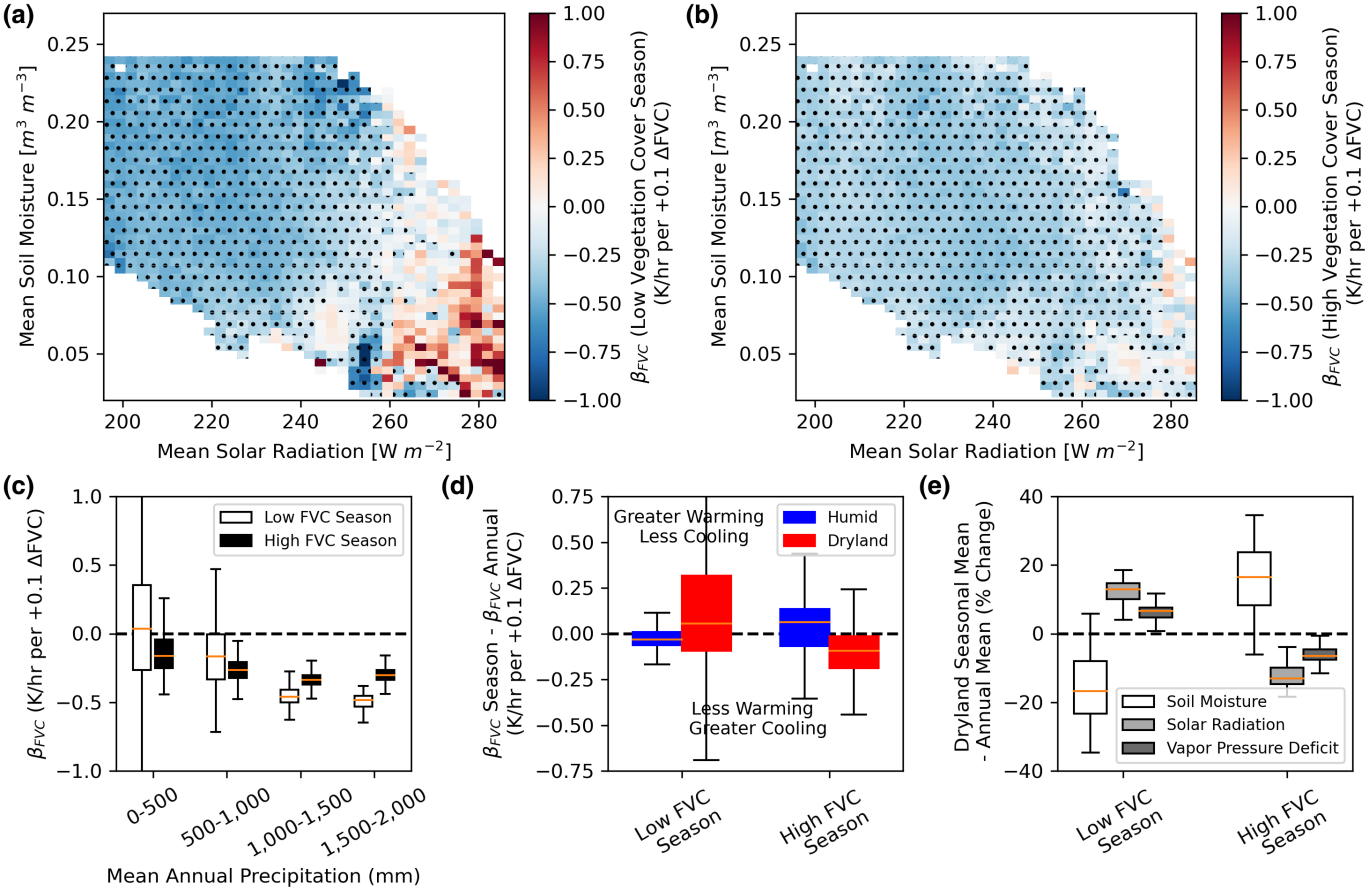
Reductions in cooling effects of African dryland vegetation observed in mean annual FVC and *d*(LST)/*dt* interactions mainly arise from seasonally drier, less vegetated conditions. (a, b) Same as Figure 2(a), but dividing into effects in the (a) less vegetated and (b) more vegetated seasons. (c) Values in (a) and (b) plotted on a rainfall gradient as divided into less and more vegetated seasons. (d) Difference between *β*_FVC_ in the given season and the overall *β*_FVC_ in Figure 1. Drylands are defined as receiving less than 500 mm of annual rainfall and humid regions are those with greater than 500 mm of annual rainfall. (e) Dryland percent difference in respective mean environmental conditions between a given season and the full year.

Our investigation of seasonal mean differences in FVC‐*d*(LST)/*dt* behavior reveals that aridity modulates vegetation‐surface energy balance interactions (Figure 3). Namely, dryland vegetation greatly loses its ability to cool the surface in times of year with lower mean vegetation cover (Figure 3a–d). These dryland vegetal cooling reductions in low vegetated seasons are linked to increased aridity. Namely, in drylands, seasons with low vegetation cover have lower mean soil moisture (−16%), higher mean solar radiation (+12%), and higher mean VPD (+6%) than the annual average (Figure 3e). In the season with more vegetation cover, these conditions switch to higher mean soil moisture, lower mean solar radiation, and lower mean VPD (Figure 3e).

Only 31% of African drylands have net vegetal cooling effects in seasons with less vegetation cover (Figure 3). This is similar to the 36% of drylands that have net vegetal cooling effects overall across the year (Figure 2), suggesting a dominant role of behavior during times of year with low vegetation cover on overall annual behavior. However, in the seasons with higher vegetation cover, cooling effects occur in 57% of drylands (Figure 3b–d). Humid tropical regions do also show some seasonal changes in behavior (Figure 3d), but with relatively consistent net cooling effects throughout the year. These patterns during the different seasons largely hold when evaluating only interannual variability with 16 years of SEVIRI data (Figure S4) as well as when evaluating behavior at seasonal timescales (Figure S7).

#### 3.2.2 Energy dissipation efficiency

**FIGURE 4:**
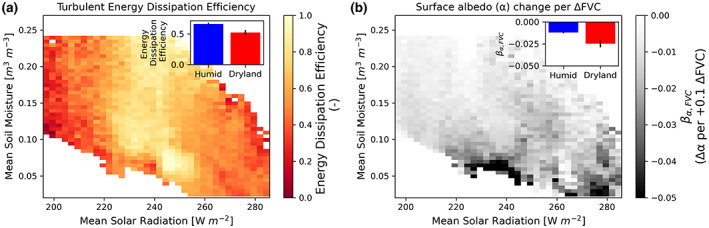
Drylands have lower overall efficiency of surface cooling via dissipating turbulent energy and amplified warming via their larger decreases in surface albedo with vegetation cover increases. (a) Estimated overall land surface efficiency of dissipating available energy (*β*_Eff_; unitless, Equation 3). (b) FVC's relationship with surface albedo (change in surface albedo per 0.1 FVC increase, Equation 4). Insets show the respective values in dryland regions (mean annual rainfall <500 mm/year) and humid regions (mean annual rainfall >500 mm/year). Error bars in insets are 95% confidence intervals based on bootstrapping of values in the respective bins showing statistically significant (*p* < .05) differences between humid and dryland environments in both cases.

Using diurnal temperature changes in Equation 3, we show that more water limited locations have less ability to cool the surface via their lower total dissipation of surface energy (Figure 4a). The diurnal changes in LST reveal that turbulent energy flux dissipation is significantly less efficient in drylands than in more humid locations (Figure 4a inset; *p* < .05).

#### 3.2.3 Surface albedo effect

Mean annual surface albedo is negatively related to FVC, not only across the study region (i.e., grasslands have higher surface albedo than forests) but also within each bin (Figure 4b). Therefore, at a given location, a unit increase in FVC will increase surface energy absorption. Furthermore, we find that drylands have over two times more surface albedo sensitivity to FVC than that of more humid regions (Figure 4b inset; *p* < .05). A 10% mean dryland FVC increase would decrease albedo by approximately 0.025 in these dry regions, resulting in an increase in mean shortwave radiation absorption of ~10 W/m^2^.

### 3.3 Assessment III: Vegetation cover's feedbacks on land surface temperature across the tropics

**FIGURE 5:**
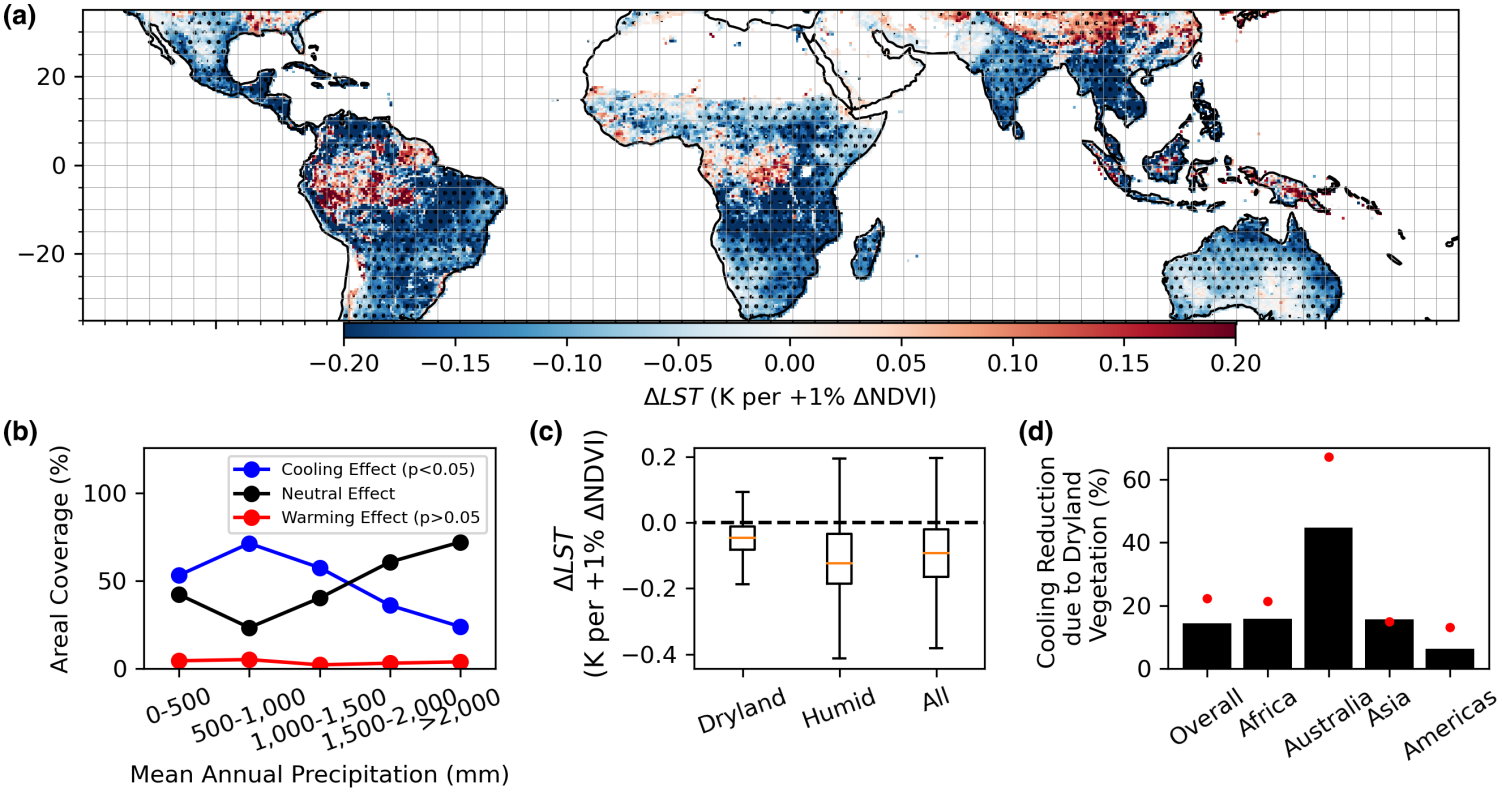
Dryland plants across the tropics tend to have reduced surface cooling effects compared to that of wetter environments. (a) Interannual effect of vegetation (NDVI) on LST determined in units of estimated change in LST per 1% increase in annual mean NDVI. Stippling indicates statistical significance of LST's partial sensitivity to NDVI (*p* < .05). Bare soil regions are removed using IGBP land cover classifications. (b) Areal coverage of statistically significant warming and cooling effects (*p* < .05) as well as neutral effects (no statistical significance; *p* > .05) binned by mean annual precipitation. (c) Distribution of values in (a) considering drylands (MAP <500 mm), humid regions (MAP >500), and all together. (d) Percent reduction in biophysical cooling due to drylands across different regions. Red dot symbols represent percent area of drylands. All regions show statistically significant reductions in overall net cooling effect reductions due to dryland vegetation (via *t*‐tests on spatial distributions of ΔLST with and without drylands included; *p* < .05).

Using satellite retrievals that extend across the tropics and are independent of datasets in Assessments I and II, we find that tropical drylands show widespread net neutral and reduced cooling effects of vegetation on LST similarly to the results in Figure 2 (Figure 5). There are similar proportions of neutral effects (52%) and net cooling effects (42%) between drylands and the remainder of tropical ecosystems (Figure 5b). Regardless, considering the pixels where the significant cooling effects were found, the median cooling effect of dryland vegetation is half the magnitude on average of that of more humid regions (Figure 5c). The cooling magnitude of dryland vegetation is also weaker in 91% of dryland pixels than the median cooling magnitude in the more humid tropics. As such, the overall cooling biophysical effect of vegetation cover on LST in drylands is weaker than that of the more humid regions across the tropics (Figure 5c).

As a result of the reduced cooling effects in drylands, drylands have an average LST change of −0.05 K per percent increase in annual mean NDVI compared to −0.1 K for more humid regions (Figure 5c). Consequently, drylands reduce the net cooling effect of tropical vegetation by 14% on average (25% by median) considering a uniform unit increase of annual NDVI across the tropics (Figure 5c). Furthermore, these reductions in net cooling effects are proportional to the total land area of drylands on a given continent; given a high land cover of drylands in Australia and Asia, drylands reduce net vegetal cooling effects even more on these continents (Figure 5d).

## 4 DISCUSSION

Observations from several satellite platforms are used to characterize vegetation's isolated effect on land surface temperature as well as attribute these effects to different surface energy balance drivers. While previous studies suggest strong net vegetative cooling effects across the tropics driven in part by semi‐arid regions (Alkama et al., 2022; Forzieri et al., 2018), we instead find that tropical dryland vegetation has relatively weaker cooling effects than in wetter environments. Such reduced vegetal cooling effects in these locations originate from reduced ability for dryland vegetation to cool the surface under water‐limitation and observed amplified warming effects due to dryland plants' strong sensitivity of surface albedo to FVC.

Our methodology has several merits providing credence to our results. (a) The results are based on observations and our methods do not require land surface modeling assumptions about competing effects of vegetation on the land surface energy balance. (b) Our methods include independent use of SEVIRI and MODIS data, as well as regressions using spatial and temporal variations that ultimately result in similar spatial patterns of results. (c) Observation‐based identification of mechanisms support these spatial patterns.

### 4.1 Question a: Does more vegetation cover net cool the surface across the tropics, especially in drylands?

We find that vegetation mainly imparts net cooling effects across the tropics. These net tropical vegetation cooling effects are expected, especially where water is ample: more vegetation cover increases the land surface's ability to evaporate rootzone moisture beyond that of bare soil and further increases evaporation through enhanced friction velocity. However, in drylands, we find that these net vegetal cooling effects become reduced and can even switch to warming effects. These findings have support from all three assessments here. Using primarily SEVIRI‐based diurnal temperature observations in Assessment I, African vegetation shows a gradient of reduced net cooling effects in more water‐limited locations that is supported by regressions using both spatial and interannual timescale analyses (Figure 2). Assessment II shows mechanisms that agree with a reduction in vegetal net cooling effects in water‐limited locations as linked to seasonal aridity conditions and higher surface albedo sensitivity to vegetation cover (see Section 4.2). Finally, repeating the analysis across the tropics with independent observations in Assessment III, a similar reduction of net vegetal cooling effects is found in drylands (Figure 5). These observed patterns and drivers ultimately inform global biophysical modeling.

While these regression approaches provide only correlative information on their own, our findings suggest that the vegetation effects on LST are causal influences for several reasons. (1) The establishment of FVC and *d*(LST)/*dt* statistical connections in Assessment I detect a more physical connection between vegetation and the energy balance, compared to LST alone (Bateni & Entekhabi, 2012; Panwar & Kleidon, 2022). (2) Our mechanistic assessment supports the spatial patterns that greater vegetal net cooling effects occur in the humid tropics (see Section 4.2). (3) While temperature effects on vegetation may confound observed patterns of vegetation‐temperature relationships, they are not expected to be dominating these relationships. Namely, cooler temperatures are not expected to proportionally increase vegetation productivity more in cooler, tropical humid ecosystems than in warmer, tropical drylands. Moreover, tropical vegetation is known to be water and/or light limited (Madani et al., 2017; Nemani et al., 2003), and thus the influence of temperature on vegetation function is likely not dominating tropical vegetation‐temperature patterns. (4) The results are similar when replacing mean *d*(LST)/*dt* with afternoon average LST, but are subdued when using morning average LST (Figure S3). This shows an effect of vegetation mechanisms on temperature, where evaporation and incoming radiation mechanisms (see Assessment II) are active after the early morning and thus influence the afternoon temperatures more than early morning temperatures. Ultimately, these arguments provide evidence for causal interpretation of vegetation effects on temperature in Assessment I as well as in Assessment III that relies on annual mean variations of afternoon LST instead of mean *d*(LST)/*dt*.

Results from complementary Assessments I and III agree, which gives credence to the results that water‐limitation inhibits vegetal cooling in the tropics. Assessment I's spatial analysis is expected to account for climatic feedbacks over longer timescales (Charney et al., 1975; Eagleson & Segarra, 1985; Green et al., 2017; Taylor et al., 2012), but has uncertainties in other spatial confounding factors that may not translate to variability in time at a location (i.e., edaphic, topographic factors, etc.). While the interannual analysis in Assessment III is based on fewer samples and does not include feedbacks, it includes effects in time at a single location and does not require space–time assumptions as in the spatial relationships in Assessment I. Together, these findings agree that aridity reduces the net surface cooling effects of vegetation in the tropics at the longer climatic timescales of biophysical feedbacks.

#### 4.1.1 Confounding effects of choice of timescale and vegetation index

We argue here that the reduced net cooling effects in tropical drylands have not been found in previous biophysical feedback investigations likely due to use of sub‐annual timescales and leaf area index.

Our results show shorter sub‐annual timescale sensitivities may confound studies of temperature‐vegetation interactions for several reasons. First, our tests using a panel regression approach to partition timescales of effects indicate that the sign of interactions between FVC and land surface temperature may switch between sub‐annual and beyond‐annual timescales, especially in drier environments (Figure S5). The results at seasonal timescales do agree that net vegetal cooling effects decrease in water‐limited locations (Figure S6), but stronger cooling effects are found at sub‐annual timescales (Figures S5 and S6). Evaluating seasonal timescales of these effects thus may be overestimating net vegetal cooling feedbacks given that FVC and *d*(LST)/*dt* seasonal cycles carry spurious relationships between variables that may inflate the strength of their relationship (Feldman et al., 2022; Tuttle & Salvucci, 2017). There is less confidence in interactions at intra‐seasonal timescales because the FVC intra‐seasonal variability has a reduced signal, occupying less than 5% of the power spectrum, which likely approaches the variability of instrument noise (Figure S6). Ultimately, we argue that these vegetation‐temperature interactions should be evaluated at beyond‐annual timescales given these aforementioned limitations at sub‐annual timescales as well as the fact that these interactions are typically interpreted in the context of long‐term change (climatic changes, land use change, etc.).

Our results suggest that use of LAI instead of NDVI and FVC may confound interpretations of vegetation's effect on the surface energy balance. Repeating Assessment III with MODIS LAI results in greater vegetal cooling effects in drylands compared to more humid locations, suggesting that tropical dryland plants are most efficient at cooling the surface (Figure S8). This change in spatial gradient of vegetation effects on LST originates primarily from the non‐linear transformation of reflectance information into LAI (Figure S9). For several reasons, we caution against using LAI for our research questions and argue that vegetation cover parameters like FVC are instead more optimal. First, interpretation of LAI variations shifts from mainly horizontal vegetation cover variations in drier, less vegetated environments to vertical structure variations in more humid, densely vegetated regions (Carlson & Ripley, 1997). Therefore, LAI variability and the resulting surface energy balance response have different meanings in each pixel, which prevents comparison of the strength of vegetation's impact on LST across space. By contrast, NDVI and FVC instead have more consistent, normalized interpretations of vegetation variability and how it influences temperature across space. Second, the amplified LAI standard deviation in humid regions (based on LAI modeling from reflectances) will inherently reduce the magnitude of vegetation's statistically modeled effect on land surface temperature. Third, our mechanistic analysis (Figures 3 and 4) and previous findings of strong warming with wet tropics deforestation (Vargas Zeppetello et al., 2020) do not support the LAI‐based finding that dryland vegetation has an increased ability to cool the surface. LAI is still suited to evaluate biophysical feedbacks within a pixel, but interpretations of LST response to LAI across space are limited for answering our questions here for these reasons. Ultimately, the FVC results are likely more indicative of horizontal vegetation structure effects (i.e. canopy coverage) and thus future work needs to also investigate the normalized impact of vertical structure effects (i.e. vegetation height and vertical leaf area variations) across space. See SI for more discussion of these arguments.

We add caution to interpreting results in wet tropical forests here, especially with the NDVI analysis in Assessment III, which show reduced statistical significance of effects (Figure 5). First, tropical forests have high cloud cover and optically opaque atmospheres that reduce optical/infrared parameter retrieval quality (Freitas et al., 2010; Göttsche et al., 2016; Trigo et al., 2021). Increased noise of the regressed variables inherently forces the regression slope to zero, switching the sign of *β*_NDVI_ in many cases. Second, NDVI saturates at high values of vegetation cover, where changes in FVC translate to proportionally smaller NDVI changes (Myneni & Williams, 1994), which leads to large erroneous *β*_NDVI_ magnitudes. Specifically, since regression slopes (i.e., *β*_NDVI_) are inversely related to the variance of their regressor (i.e., NDVI variance), an underestimated vegetation cover variance with NDVI will inflate *β*_NDVI_. Both effects are confounding *β*_NDVI_ values in wet tropical forests because the wet tropical forest *β*_NDVI_ values are generally not statistically significant, typically have large magnitude ranges, and tend to switch sign spatially within the same regions. Finally, previous studies found consistently strong cooling effects of wet tropical forests (Alkama & Cescatti, 2016; Mahmood et al., 2014; Silvério et al., 2015). Nevertheless, our spatial analysis in Assessment I using FVC does detect net vegetal cooling effects in Africa's wet tropical forests (Figure 2).

### 4.2 Question b: Which surface energy balance mechanisms are responsible for the observed spatial pattern of vegetal effects on surface temperature in the tropics?

Using an observation‐only identification of drivers in Assessment II, we find that drier environments have reduced net vegetal cooling effects because of their plants' reduced ability to cool the surface under higher aridity (Figure 3), their similarly reduced cooling with lower land surface energy dissipation efficiency (Figure 4a), and their vegetation cover's proportionally larger solar radiation absorption per unit increase in vegetation cover (via albedo effects) (Figure 4b). Evidence of these drivers gives additional credence to causal influences of FVC on LST as well as the spatial gradient of these interactions from wet to dry tropical environments in Figure 2.

In water‐limited locations, the loss of net vegetal cooling effects in seasons when vegetation cover is reduced is likely due to vegetation interactions with aridity (Figure 3). A large reduction in soil moisture will reduce transpiration in these dry locations, with less water available to supply leaf gas exchange as well as lower stomatal conductance (Katul et al., 2012; Manzoni et al., 2013; Rigden et al., 2020; Sperry et al., 2016). Such a moisture driven reduction in transpiration is expected based on observed strong positive control of soil moisture on gross primary production and stomatal conductance (Haverd et al., 2017; Novick et al., 2016; Short Gianotti et al., 2019). Larger VPD and incoming radiation can act to further reduce leaf stomatal conductance (Jarvis, 1976; Medlyn et al., 2011). As such, dryland vegetation can tend to exhibit reduced net cooling effects under more arid conditions because additional vegetation cover will marginally increase transpiration, but will absorb more radiation with reduced surface albedo. Weaker net cooling in drylands than humid environments during the wetter, vegetated season is further evidence of this aridity control (Figure 3b,c). Phenology may additionally contribute to these large seasonal changes with lower vegetal transpiration cooling during drier seasons (Adole et al., 2018).

The finding of reduced energy dissipation efficiency in drylands shows additional, independent evidence that surface cooling from fluxes is reduced in water‐limited environments (Figure 4a). In dry, warm locations, this ultimately suggests lower latent heat fluxes, the most efficient energy flux dissipation method in these hot environments (Bateni & Entekhabi, 2012). While the dissipation efficiency metric (Equation 3) does not isolate only vegetation effects from bare soil contributions, we argue that lower vegetation cover is largely reducing energy dissipation efficiency in dryland locations. Vegetation cover can increase wind friction velocity and thus increase surface conductance of turbulent energy fluxes. Such energy dissipation effects are reduced in arid regions with shorter, and sparser vegetation (Panwar et al., 2020). In addition, less vegetation cover results in less ability to evaporate and cool the surface using deeper rootzone moisture supply than that of bare soil. There may be additional effects of wind stilling that occur in drylands where more vegetation slows the near surface wind, creating more resistance to energy dissipation (Zeng et al., 2018).

In general, we find a negative response of surface albedo to more vegetation cover, which is expected given vegetation's ability to decrease shortwave reflectivity through its absorbing colors and canopy multiple scattering (Figure 4b). However, increases in dryland vegetation cover tend to have amplified reductions in surface albedo, contributing to proportionally greater warming effects (Figure 4b). Such a non‐linear spatial relationship between vegetation indices and surface albedo has been observed previously, and is likely due to interactions of vegetation color contrast with bare soil as well as efficiency of canopy shortwave scattering in grasses and shrubs (Fuller & Ottke, 2002; Pang et al., 2022). Specifically, remote sensing emissivity observations reveal brighter soils in drier environments, which can increase the vegetation‐soil reflectivity contrast (Peres & DaCamara, 2005) where additional vegetation cover can greatly change the surface albedo compared to humid environments. Previous work has also found that strong albedo effects can drive the interaction between vegetation and LST in drylands, such as those in Africa (Chen et al., 2020).

Our investigation of mechanistic drivers is non‐exhaustive, and we speculate that other mechanisms such as leaf‐level photosynthetic strategies may be partly driving results. Namely, we found that grasses have reduced net cooling effects compared to woody vegetation (Figure 2d). These warm tropical grasslands are typically dominated by C_4_ species compared to C_3_ woody species in humid ecosystems (Still et al., 2003). C_4_ species have a higher water use efficiency (Edwards et al., 2010; Osborne & Sack, 2012), which is a driver of observations that grasses have reduced cooling through transpiration and larger sensible heat fluxes (Panwar et al., 2020; Sellers et al., 1992). Therefore, while grassland reduction in cooling is partly driven by water availability and surface albedo considerations, the spatial gradient of net vegetal cooling in the tropics may be accentuated by existence of more C_4_ species (that have lower transpiration cooling) in grasslands and savannas dominating drier ecosystems.

### 4.3 Question c: To what degree does tropical dryland vegetation net warm or cool the land surface compared to the remainder of the vegetated tropics?

We find that under a unit fractional increase of NDVI across the tropics, there is an overall net cooling effect of vegetation based on Assessment III (Figure 5c). However, tropical drylands reduce the mean tropical biophysical net cooling effect by 14% (25% by median) (Figure 5c). This magnitude of reduction of the cooling feedback is partly controlled by dryland areal coverage (Figure 5d). As such, if climate change causes aridification in the tropics (Berg & McColl, 2021; Huang et al., 2016; Lian et al., 2021), then more subdued vegetal cooling can be expected based on our results. The fractional dryland reduction in cooling effects is likely even larger considering uncertainties with wet tropical forests results noted in Section 4.1.1. When removing wet tropical forests from the analysis, tropical drylands reduce the mean tropical biophysical net cooling effect by 20% (26% by median).

To evaluate the proportional contribution of net vegetal cooling effects of all tropical locations to the average, this simple experiment assumes that all tropical land surfaces show the same proportional greening. Studies agree there are widespread global greening trends as driven by CO_2_ fertilization, climate change, and land use change (Winkler et al., 2021; Zhu et al., 2016). Since greening would influence surface temperature, the absolute magnitude of this biophysical feedback ultimately depends on the spatial pattern of greening (Alkama et al., 2022). However, our question addresses the relative impacts of vegetation cover on land surface temperature (i.e., biophysical feedback sensitivity) to compare effects of different biomes across the tropics, rather than the actual, predicted change in temperature from greening‐related feedbacks. Our findings thus do not depend on the rates of greening.

While our results agree that tropical vegetation creates net cooling effects, our results ultimately disagree with previous studies that find strong dryland surface temperature sensitivity to vegetation in arid ecosystems, especially in the tropics (Alkama et al., 2022; Forzieri et al., 2020, 2018). Instead, we find that greening in tropical arid environments, and broadly water‐limited ecosystems, provide relatively weaker cooling feedbacks. These previous studies are ultimately based on LAI and daily timescale variability. We identify in our assessments that these methodological choices can overestimate dryland vegetation's ability to net cool the surface relative to other locations under multi‐year greening.

These results show that tree planting should cool surfaces where water is ample in the tropics. However, there may be less efficient cooling effects if planting new vegetation in water‐limited locations, unless a more optically reflective and/or deeper rooted plant species is chosen (Jackson et al., 2008). Careful attention to land management solutions in drylands is recommended (Rohatyn et al., 2022), especially for efforts like the Green Great Wall initiative which aims to plant vegetation to slow the spread of desertification in the Sahel (Duveiller et al., 2018).

## CONFLICT OF INTEREST

The authors declare no conflict of interest.

## Supporting information


Data S1

## Data Availability

The data that support the findings of this study are available in Zenodo at 10.5281/zenodo.7117263. These data were derived from the following resources available in the public domain: EUMETSAT LSA SAF temperature, fraction of vegetation cover, and solar radiation data are available from https://landsaf.ipma.pt/en/. The MT‐DCA soil moisture dataset retrieved from SMAP is freely available at https://doi.org/10.5281/zenodo.5579549. AIRS data are available at https://airs.jpl.nasa.gov/data/get‐data/standard‐data/. The MODIS NDVI product can be obtained from https://modis.gsfc.nasa.gov/data/dataprod/mod13.php. The MODIS LST product can obtained from https://lpdaac.usgs.gov/products/myd11c2v006/. CHIRPS data are available at https://www.chc.ucsb.edu/data/chirps. CPC data are available at https://psl.noaa.gov/data/gridded/data.cpc.globalprecip.html. MERRA2 data can be accessed at https://gmao.gsfc.nasa.gov/reanalysis/MERRA‐2/data_access/.
