# Supplementary material for: Tropical surface temperature response to vegetation cover changes and the role of drylands
Source: Glob Chang Biol. 2022 Oct 11;29(1):110–25. doi: 10.1111/gcb.16455 (PMC10092849; doi:10.1111/gcb.16455)
Supplement: Supplementary file 1 — Data S1 [file GCB-29-110-s001.docx]

**Supplemental Information**


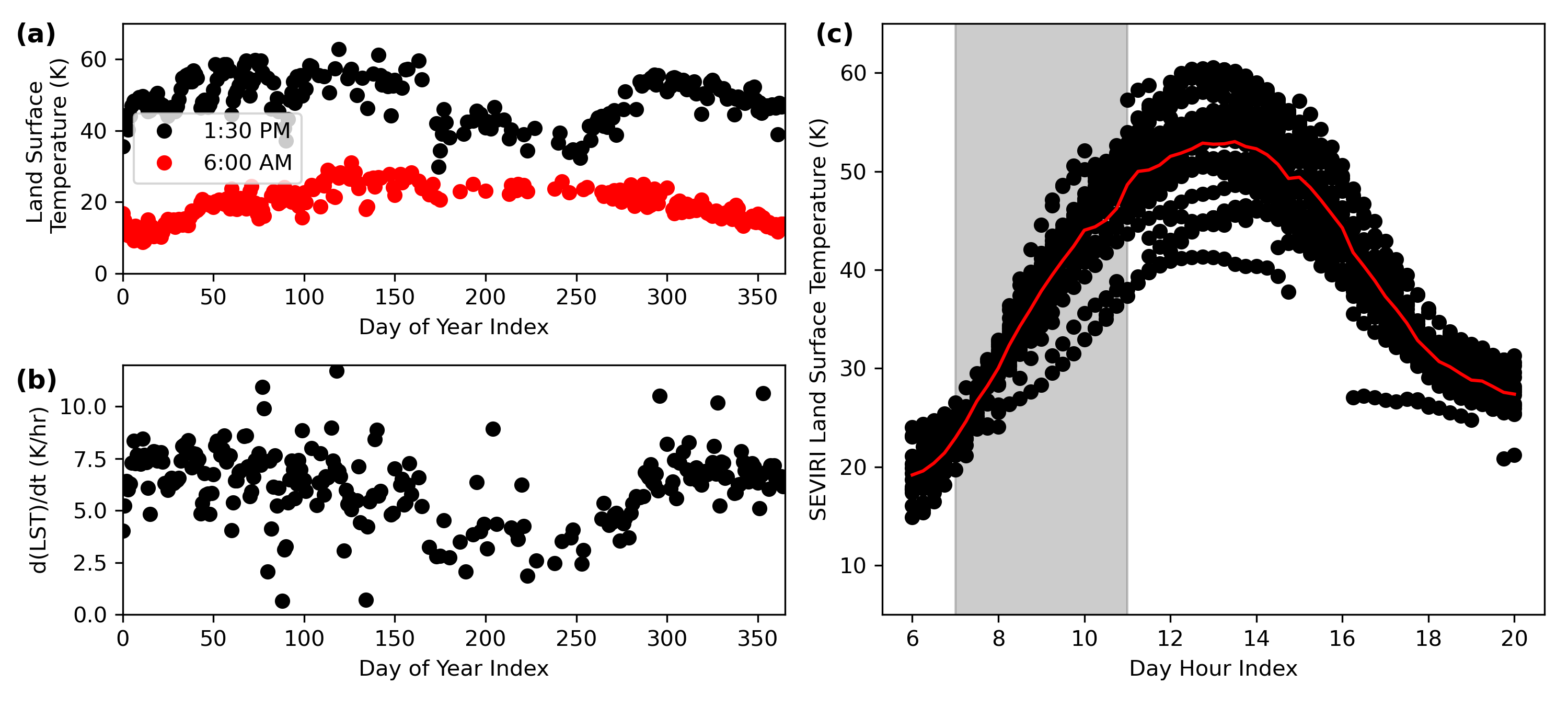


Figure S1. SEVIRI diurnal land surface temperature retrievals at a representative pixel in the Sahel in Africa in 2018 (latitude = 12.2°N, longitude = 16.1°E). **(a)** Daily LST at 1:30 PM and 6:00 AM, near the maximum and minimum daily temperatures, respectively. **(b)** Median daily d(LST)/dt computed based on the procedure described in Section 2.3.1 with values between 7 am and 11 am. **(c)** The diurnal cycle of all days within March 2018. The grey shading are hours between 7 am and 11 am when the LST growth rate is approximately linear. The median diurnal cycle for all values in the month is shown in the red line.


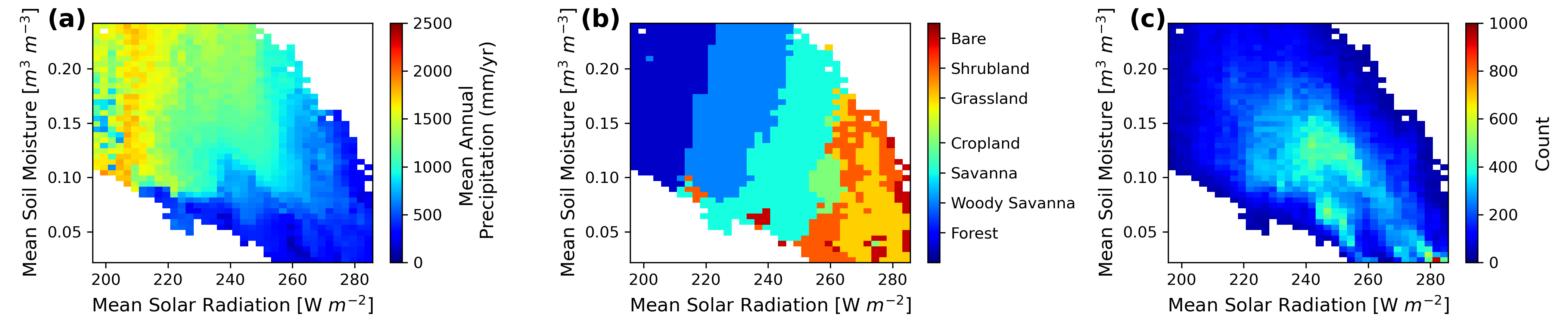


Figure S2. Summary characteristics of the binned pixels with **(a)** mean annual precipitation, **(b)** dominant IGBP land cover classification, and **(c)** 9km pixel EASE2 pixel count within each bin.


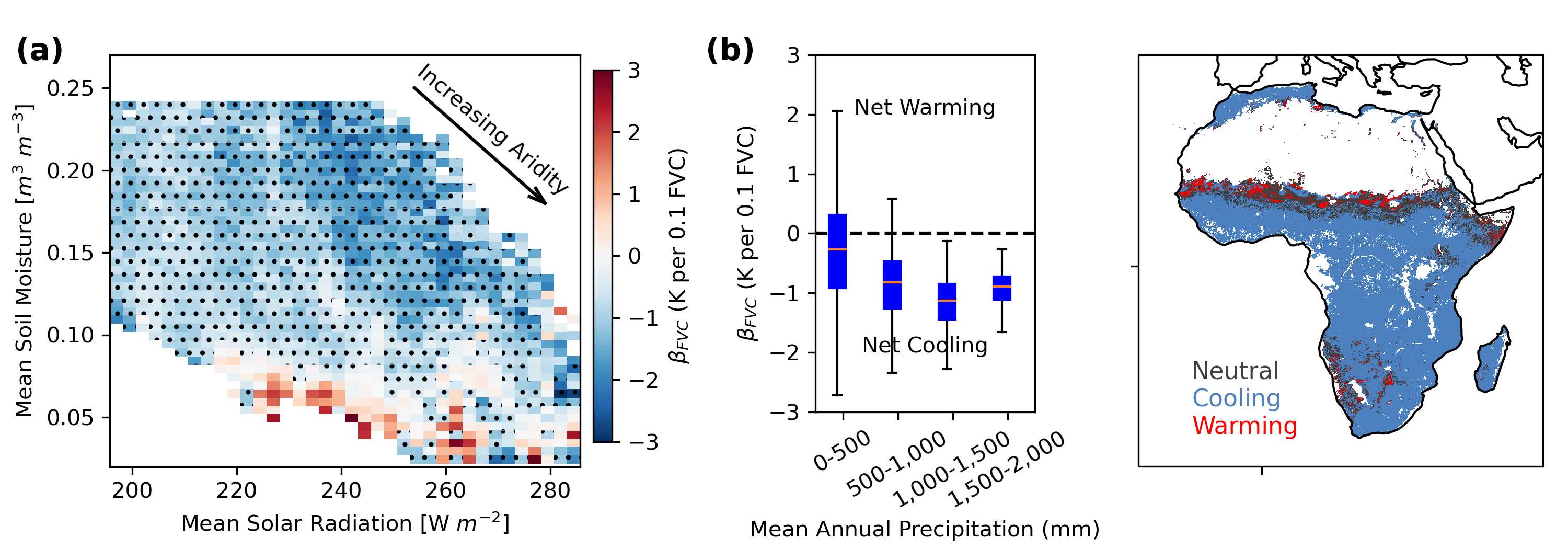


Figure S3. Same as Fig. 2, but evaluates effects of FVC on mean annual land surface temperature at 1:30pm. Effects at 6:00am are muted (not shown).

***Interannual Variability Effects and Effects in Different Seasons***

We conducted two tests to determine explicit interannual timescale relationships within pixels to assess whether the spatial relationships in Eq. 1 are indeed occurring in time. In both tests, we estimate d(LST)/dt using dT or the diurnal temperature amplitude (difference in 1:30pm and 6:00am land surface temperatures). dT is used in place of d(LST)/dt due to computational constraints in working with multiple years of 15-minute time-step LST measurements at a SEVIRI grid scale of approximately 3km. In the first test, four years of concurrent LST, FVC, SM, and Rs data when SMAP and SEVIRI overlap (2015-2019) are used. The pixels are binned spatially as in the method in Eq. 1 and the annual means for each of the four years of d(LST)/dt, FVC, SM, and Rs are computed. Within each bin, four pixels are chosen at random to assess the annual averaged dT and FVC relations over the four years while controlling for SM and Rs as in Eq. 2. This process is repeated in a Monte-Carlo framework in 100 iterations and the median $\beta_{FVC}$ is computed for each bin. In the second test, each pixel is individually assessed using Eq. 2 for interannual relationships between FVC and d(LST)/dt means over 16 years from 2004 to 2019. Since soil moisture is not available during this time period, total annual precipitation from CHIRPS is used in place of mean soil moisture.

The second test more directly evaluates interannual variability effects of FVC on d(LST)/dt. However, it includes uncertainty in using precipitation which is not as direct of an indicator of surface water availability as soil moisture and is prone to error in a data sparse region (Africa) where CHIRPS relies on in-situ station data. The first test explicitly controls for soil moisture, but requires mixing space and time in only using four years of data.


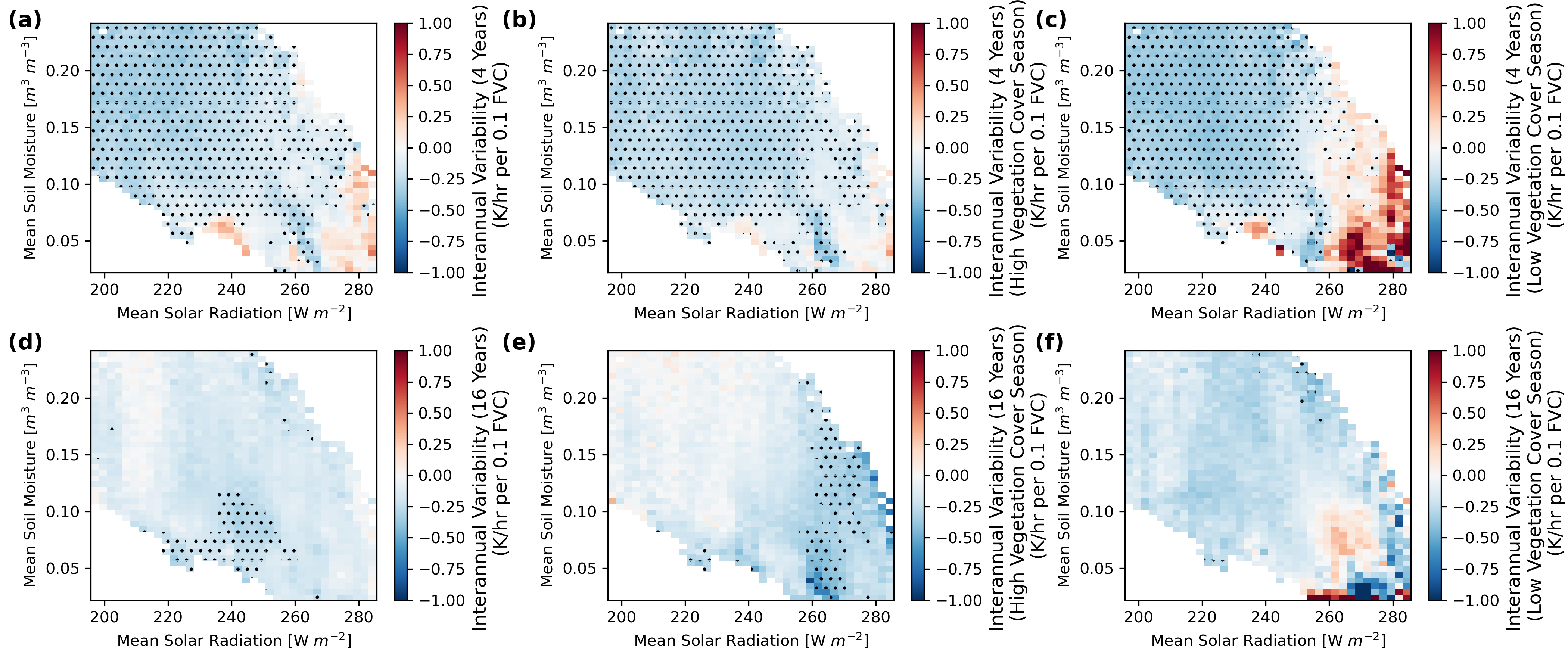


Figure S4. FVC effect on d(LST)/dt (estimated using dT) at interannual timescales. **(a-c)**: Interannual variability of dT assessed over 2015-2019 while controlling for soil moisture annual mean changes in a given pixel using SMAP. **(d-f)**: Interannual variability of dT assessed over 2004-2019, but controlling for mean moisture availability using CHIRPS annual precipitation.

***Spatial and temporal effects combined in pooled model***

We test whether different vegetation-temperature interactions occur at sub-annual and beyond annual timescales. A pooled regression model is used where a dummy variable is added to Eq. 2:

$d(LST)/dt=\beta_{0}+\beta_{FVC\_Seasonal}FVC+\beta_{SM\_Seasonal}SM+\beta_{Rs\_Seasonal}Rs+\gamma_{FVC\_Spatial\_50th}{FVC}_{Mean Indicator}+\epsilon$ (S1)

In this case, the same format as Eq. 1 is used by binning pixels that have nearly identical Rs and SM means. However, the full time series is used in each pixel. The difference is that the dummy variable (FVC_Mean Indicator_) isolates annual mean FVC effects from the seasonal FVC effects. FVC_Mean Indicator_ takes a value of zero (one) if the given pixel has a below (above) average annual mean FVC. Therefore, negative values of γ_FVC Spatial 50th_ suggest that having an above average mean annual FVC has a surface cooling effect (decreases d(LST)/dt in the annual mean). This approach provides an alternative means to estimate longer annual timescale effects of FVC on d(LST)/dt, while explicitly controlling for effects of seasonal timescale interactions. It can also detect whether opposing sign FVC effects are occurring at seasonal ($\beta_{FVC\_Panel}FVC$) and longer timescales ($\gamma_{FVC\_Spatial\_50th}$). Note that since γ_FVC Spatial 50th_ is the coefficient of a dummy variable and not a continuous variable, it is interpreted as a statistical test of its sign rather than providing a mean sensitivity between FVC and d(LST)/dt (as in Eq. 1). Therefore, magnitudes of γ_FVC Spatial 50th_ and β_FVC_ cannot be directly compared.

A multiple regression approach is applied to also evaluate seasonal and intra-seasonal relationships on a per-pixel basis:

$dLST/dt=\beta_{0}+\beta_{FVC}FVC+\beta_{SM}SM+\beta_{Rs}Rs+\varepsilon$ (S2)

For seasonal timescales, the raw time series are used. Intra-seasonal relationships are tested applying Eq. S2 to each deseasonalized variable, by subtracting a 30-day climatological moving average window from the time series to obtain anomalies (Feldman et al., 2020). For all pixels in the same bins as those used in the primary analysis, the pixel-wise intra-seasonal and seasonal β_FVC_ values (from Eq. S2) are averaged for comparison.


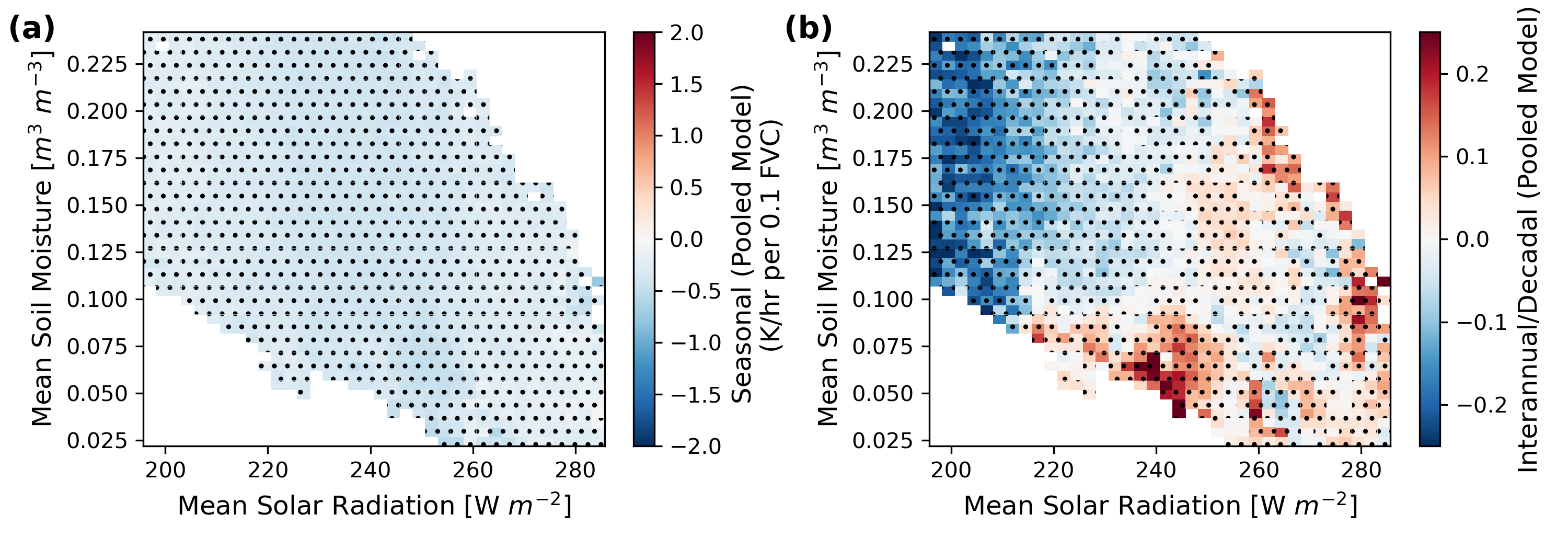


Figure S5. Panel regression tests show that, simultaneously, mean annual FVC-d(LST)/dt relationships can be positive while FVC-d(LST)/dt seasonal relationships can be neutral or negative, especially in more water-limited locations. Relationships between mean annual FVC and d(LST)/dt in both time and space while controlling for surface soil moisture and light availability using a conditioning approach. **(a)** Relationship in time representing seasonal variations (β_FVC Temporal_). **(b)** Relationship in space representing beyond-annual timescale variations (γ_FVC Spatial 50th_). Stippling indicates a statistically significant (p<0.05) FVC and d(LST)/dt relationship. Each bin includes pixels with nearly identical mean soil moisture (±0.0025m^3^m^-3^) and mean incoming solar radiation (±1.25Wm^-2^).

Figure S6. Same as Fig. 2a, but includes intra-seasonal and seasonal variability.


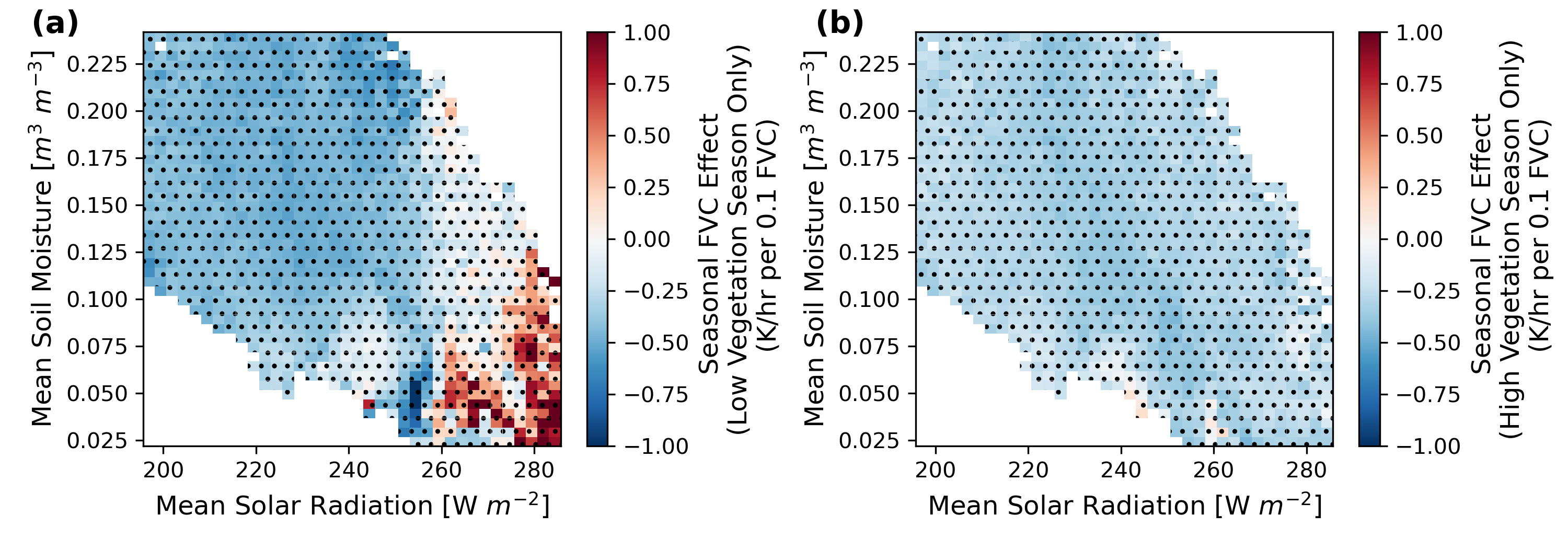


Figure S7. Seasonal timescale effect of FVC on d(LST)/dt in both the **(a)** lower and **(b)** higher vegetated seasons of the year.

***Use of Normalized Vegetation Parameters Compared to Leaf Area Index***

Repeating the analysis with LAI instead of NDVI results in the appearance of dryland tropical environments having the strongest cooling effects with progressively more subdued effects in more humid locations (Fig. S8). This is the result of effects of LAI interannual variability and FVC interannual variability on LST diverging progressively from dry to humid environments (Fig. S9). We argue here that NDVI and FVC are more optimal parameters to assess spatial gradients of effects of vegetation on the surface energy balance and LST. We outline several reasons for this argument.

First, we find that the way in which LAI is modeled describes the difference in results from those of NDVI and FVC in our study. As such, there is a potential artifact with modeling LAI as nonlinearly increasing with NDVI and FVC. We repeat the analysis using SEVIRI LAI and FVC in Africa to determine their respective effects on LST (Fig. S9), where SEVIRI LAI is explicitly modeled directly from FVC with an exponential function and clumping parameters (García-Haro and Camacho, 2014). MODIS LAI and NDVI are produced from separate algorithms, but we expect that the test with SEVIRI LAI and FVC will isolate the direct effect of modeling LAI on results of the study and describe the differences in results with MODIS LAI and NDVI in Fig. S8. Namely, while FVC and LAI are approximately linearly related in less densely vegetated regions (Carlson and Ripley, 1997), unit changes in FVC are directly translated to result in proportionally larger LAI changes in more densely vegetated locations within the SEVIRI algorithm (García-Haro and Camacho, 2014). As such, the interannual variability of LAI increases in wetter locations while FVC (as well as NDVI) interannual variability tends to be largest in transitional climates with reductions in dry and wet environments due to being a bounded variable (not shown). As a property of least squares regression, the regression slopes of vegetation indices predicting LST (Eqs. 1 and 2) are an inverse function of the vegetation index’s standard deviation. Therefore, with increasing vegetation density, LAI-based assessments of effects on LST inherently force their perceived impact on LST toward zero because of increasing LAI standard deviations. This can be seen in Fig. S9 where SEVIRI LAI’s effect on LST shows progressively subdued cooling effects in more humid regions, solely due to the way that LAI is modeled from FVC. This behavior is not observed in the FVC-based analysis. Therefore, the difference in effects of LAI and FVC on LST are amplified in humid regions considering that because NDVI and FVC are bounded, their standard deviations reduce slightly in more humid environments while LAI standard deviations increase in these regions. We suggest that with similar modeling of MODIS LAI from MODIS reflectances, this LAI standard deviation spatial pattern analogously explains the difference in results between MODIS LAI and MODIS NDVI (Compare Fig. S8b, S8c). A question arises whether modeling LAI from FVC captures the true holistic effect of vegetation on the surface energy balance LST. With the subsequent reasons, we instead argue that use of LAI presents confounding factors that would bias an analysis that addresses our research questions here.

Second, we argue that LAI is not as optimal as NDVI and FVC for our research questions because LAI is not a normalized parameter when interpreting spatial gradients of its effects on LST. Namely, changes in LAI can represent both horizontal to vertical vegetation variability, hindering consistent interpretation of LAI-based vegetation effects on the surface energy balance across climates (Carlson and Ripley, 1997). While NDVI and FVC represent the vegetation cover and greenness across biomes (i.e., horizontal structure), LAI shifts its representation from horizontal to vertical vegetation structure from less to more vegetated biomes – leaves are added more vertically than horizontally in wooded, wetter pixels while they tend to be added more horizontally in drier pixels. Therefore, changes in LAI and thus a regression coefficient with LAI will have different interpretations in each pixel - when assessing vegetation’s impact on LST, the way in which additional leaves are added to a pixel matter for their impact on LST. Leaves added horizontally (i.e., new plants) will change the pixel surface albedo via altered vegetation and bare soil fractions, while the same leaves added vertically may greatly increase surface turbulent flux conductance. Therefore, due to no constraint on structure in LAI, differences in LAI’s impact on LST across different pixels may indicate the strength of the energy balance mechanism that is altered (vegetation color, bare soil-plant contrast, transpiration, etc.) related to how leaves change within a pixel (horizontal density or vertical density) rather than vegetation’s normalized, integrated influence on LST. By contrast, FVC and NDVI provide more normalized parameters to evaluate effects on land surface temperature because their unit changes consistently translate to similar vegetation structure changes (i.e., vegetation coverage and color contrast with bare soil) and thus similar surface energy balance mechanisms across pixels. As such, LST responses to FVC and NDVI can directly be compared across pixels and the regression analysis should reflect LST’s response to horizontal plant structure information (i.e., greenness and vegetation cover variations). Vertical vegetation structure variations across space should be investigated in the future using normalized indices that account for vegetation height and leaf biomass with height, independent of horizontal vegetation structure information. Such detailed variations will determine whether, for example, vegetation cover or vegetation height strengthen surface cooling effects.

As an example of LAI-based differences in results, we find that in more humid environments NDVI variations have a stronger cooling effect on LST compared to drier environments while LAI variations in more humid environments have a weaker cooling effect on LST compared to drier environments (Fig. S8). We argue, based on the aforementioned reasoning, that the LAI-based result likely does not indicate that vegetation overall in humid environments has a weaker effect on land surface temperature. Rather, it instead means that the vertical vegetation properties that dominate the LAI signal in wetter environments may have a smaller effect on LST than the horizontal considerations of bare soil-vegetation contrast represented more by FVC and NDVI. Stated alternatively, the LAI-based results suggest that adding leaves in more humid locations presents more marginal returns in land surface cooling than in drylands. However, this result only reflects the nature in which vegetation growth tends to create additional leaves on the land surface: vertically in more humid biomes and horizontally in drier biomes. Therefore, in order to directly compare magnitudes of vegetation influence on LST between a humid and dry region, one would need to isolate the LST response to the vertical (or horizontal) addition of leaves in both humid and dry regions, which LAI alone does not allow. Analogously, FVC (and NDVI) provides the same horizontal vegetation variation information in each pixel, which allows a normalization to directly compare LST responses to their vegetation index variations across space. LAI trends can be used concurrently with LST sensitivities to LAI to infer biophysical feedbacks at a location as has been done in previous work. However, LAI-based vegetation-LST interactions are dependent on the vegetation structure and are challenging to compare pixel to pixel across space.

Third, the fact that LAI’s effect on LST approaches zero in more humid environments is not supported by our mechanistic evaluations that explain why cooling effects would decrease in drier environments (Figs. 3 and 4). It is also not supported by many studies finding a strong cooling effect of vegetation in tropical forests with consequently strong warming under deforestation (Vargas Zeppetello et al., 2020).

Fourth, LAI tends to be the most modeled parameter of the vegetation indices compared to FVC and NDVI and is thus expected to have the most uncertainty in its variations.

Therefore, for these reasons, we argue that the use of FVC or NDVI may be better suited to evaluate gradients in vegetation’s effect on land surface temperature.


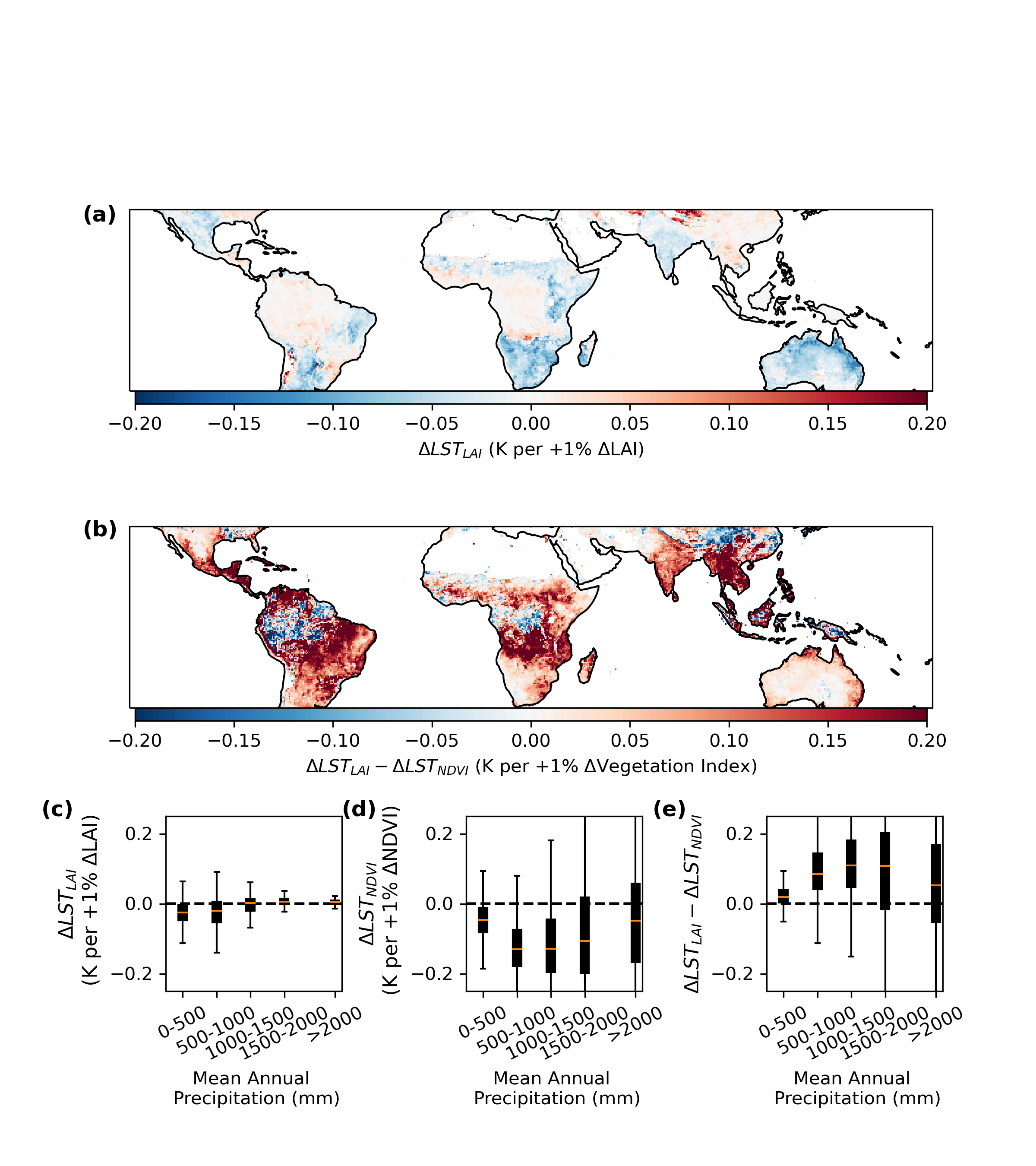


Figure S8. **(a)** Same as Fig. 5a but using MODIS LAI instead of MODIS NDVI. **(b)** The difference in LST changes based on 1% increases in mean LAI and LST changes based on 1% increases in mean NDVI. Plotting values along a rainfall gradient for LST changes based on **(c)** 1% increases in mean LAI (based on **(a)**), **(d)** 1% increases in mean NDVI (based on Fig. 5a), and **(e)** their normalized difference (based on **(b)**).


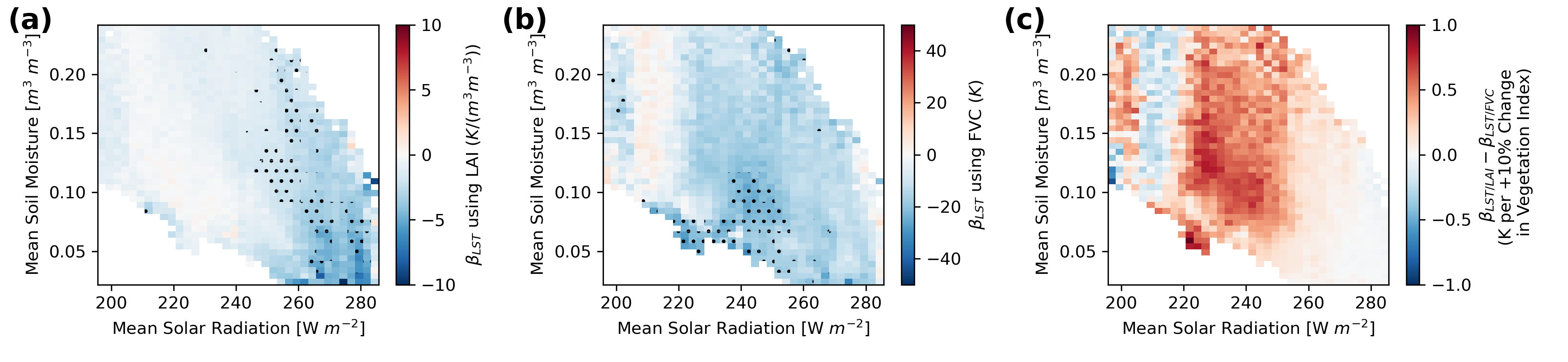


Figure S9. **(a)** Effect of SEVIRI LAI on SEVIRI LST at interannual timescales. **(b)** Same as **(a)** but using SEVIRI FVC. **(c)** The normalized difference of effects on LST between SEVIRI LAI and SEVIRI FVC. Positive values indicate that LAI suggests reduced cooling or stronger warming of the surface than FVC does. Both effects on LST have units of K per 10% increase in annual mean vegetation index where values in **(c)** were normalized by multiplying values in **(a)** and **(b)** by 0.1 times the annual means of LAI and FVC, respectively.
